# Changes in Psychological Inflexibility and Intimate Partner Violence Among Men in an Acceptance and Commitment Therapy-Based Intervention Program

**DOI:** 10.3390/bs15030317

**Published:** 2025-03-06

**Authors:** Amie Zarling, Meg Berta, Ashlyn Miller

**Affiliations:** Department of Human Development and Family Studies, Iowa State University, 1358 Palmer Building, Ames, IA 50011, USA; mmberta@iastate.edu (M.B.); ashmil@iastate.edu (A.M.)

**Keywords:** domestic violence, intimate partner violence, batterer intervention program, corrections, psychological flexibility

## Abstract

Understanding the mechanisms of change in batterer intervention programs (BIPs) is essential for advancing effective interventions for intimate partner violence (IPV). This study evaluates changes in psychological inflexibility and IPV behaviors among men participating in an acceptance and commitment therapy (ACT)-based BIP for men court-mandated to domestic violence classes. Participants (*N* = 56) were assessed across three time points using validated measures of psychological inflexibility and IPV behaviors. Findings revealed significant improvements in psychological inflexibility and corresponding reductions in IPV behaviors over the intervention period, with medium to large effect sizes. Psychological inflexibility at the final assessment predicted IPV outcomes, accounting for 15% of the variance, even after controlling for baseline IPV, age, and race. These results highlight psychological inflexibility as a potential mechanism of change in IPV interventions. Despite this study’s strengths, including its longitudinal design and rigorous analyses, limitations such as a small sample size and lack of a control group warrant further investigation. This study underscores the potential of ACT-based interventions to reduce IPV by targeting psychological inflexibility and offers insights for refining BIPs to enhance their impact.

## 1. Introduction

Intimate partner violence (IPV) is a complex social and behavioral issue, and there are various different philosophies and approaches to intervening with the men who have perpetrated gender-based violence (Murphy et al., 2019). Regardless of the theoretical framework or model guiding an intervention, such programs typically aim to foster internal changes in participants by targeting specific underlying processes that drive outcomes. Most evaluations of these programs–often called batterer intervention programs (BIPs)–acknowledge their modest efficacy while often offering little evidence to support proposed alternatives. Studies and meta-analyses generally show that BIPs reduce partner violence reoffending with small-to-medium effects, though results vary by outcome type and research design (e.g., Babcock et al., 2024; Cheng et al., 2021). Methodological issues, such as high attrition and limited success indicators, remain common, and few alternatives to BIPs, such as incarceration or probation-only approaches, are effective. Thus, while BIPs should continue, there is a pressing need for improved strategies to enhance their impact and more robust evaluation to support their growth.

One potential avenue to improve BIPs is to examine the processes or mechanisms through which outcomes are achieved. Among the limited studies that have examined these mechanisms, few have been able to determine for whom, under what conditions, and why BIPs are effective, when they do work. A review of quantitative research by Velonis et al. (2020) identified only six studies that included sufficient detail to assess program processes, with self-reflection and empathy emerging as potential predictors of success in certain contexts.

Leading experts in both criminal justice (e.g., Howard & van Doorn, 2018; Serin et al., 2013) and psychology (e.g., Kazdin, 2009) have consistently emphasized the need for rigorous studies on change to establish causal relationships between specific program components and outcomes. Current gaps in understanding the processes of change hinder progress in the field, as it remains unclear which elements of BIPs contribute most to successful reductions in IPV and criminal recidivism. These critical program components, often referred to as the “black box” of programs (Latessa, 2004), represent the key elements that drive meaningful behavioral change. Understanding this “black box” allows practitioners and researchers to develop a blueprint for effective interventions by identifying and prioritizing the content and processes that are most impactful (Kroner & Yessine, 2013). When a program aligns with its underlying theory of change, we gain insights into the relationships between changes in dynamic risk factors and reoffending outcomes, bringing us closer to unraveling the “black box.” This understanding is essential for translating research findings into practical applications within correctional settings. Without identifying the mechanisms that make treatments effective, implementation efforts are likely to fall short. As Serin et al. (2013) aptly put it, the field continues to search for the “Holy Grail in assessing offender change”.

### 1.1. Psychological Flexibility

Psychological flexibility is defined as staying present and open to experiencing thoughts, emotions, and sensations while acting in ways that align with one’s core values, even in the face of challenges or distress. Individuals who are psychologically flexible can navigate challenging emotions and thoughts with openness, positively influencing other psychological processes and supporting their healthy expression. In contrast, psychological inflexibility—marked by an over attachment to thoughts or the avoidance of distressing experiences, or even excessive clinging to seemingly positive ones—can have harmful effects (e.g., Hayes et al., 2012). Psychological (in)flexibility is believed to be directly modifiable through targeted interventions such as acceptance and commitment therapy (ACT; Hayes et al., 1999). ACT specifically focuses on increasing psychological flexibility and reducing psychological inflexibility as a means of reducing maladaptive behaviors.

A growing body of research highlights a negative association between psychological flexibility and aggression, with psychological inflexibility processes demonstrating a stronger predictive relationship with aggression (Berkout et al., 2019; Donahue et al., 2024). Studies examining individual flexibility and inflexibility processes provide a more nuanced understanding of these relationships. Among inflexibility processes, experiential avoidance—the avoidance of unwanted internal experiences, such as jealousy—is the most extensively studied. Multiple studies have shown a link between higher levels of experiential avoidance and increased aggression (e.g., Bell & Higgins, 2015; Shorey et al., 2014; Theodore-Oklota et al., 2014). For example, Grom et al. (2021) found that alcohol use exacerbated IPV perpetration among individuals with high experiential avoidance but not among those with low experiential avoidance.

To further examine the role of experiential avoidance in IPV, LaMotte et al. (2025) used a scenario-based approach, presenting 74 men enrolled in a BIP with emotionally challenging relationship vignettes to assess their anticipated emotional reactions, motivation to reduce those emotions, and likelihood of engaging in abusive or non-abusive behaviors. Data were collected through structured interviews and validated measures of experiential avoidance, with analyses examining the relationships between emotional intensity, motivation, and behavioral intentions.

This study found that the intensity of negative emotions anticipated by participants, their motivation to reduce those emotions, and their belief that abusive behaviors would alleviate emotional distress significantly predicted their likelihood of engaging in abusive responses. Notably, the expectation that abusive actions would repair negative emotional states was the strongest and most unique predictor of abusive behavioral intentions, while none of these variables significantly predicted non-abusive responses. These findings suggest that experiential avoidance plays a critical role in partner abuse by driving abusive behaviors as a means to escape negative emotions.

Garofalo et al. (2020) examined relationships among mindfulness, emotion regulation, and aggression dimensions (e.g., verbal and physical aggression, anger, and hostility) in both violent offender and community samples. Their findings demonstrated that greater impairments in mindfulness and emotion regulation were associated with increased levels of aggression across both samples. Additionally, emerging evidence suggests that emotional acceptance is negatively associated with aggressive behavior (e.g., Zhang et al., 2023). A systematic review by Navas-Casado et al. (2023) synthesized findings from 104 studies examining the relationship between emotion regulation and aggression. The review revealed that maladaptive strategies, many of which serve as avoidance functions (e.g., rumination), are positively associated with aggression, while adaptive strategies, such as mindfulness, are negatively associated. This relationship between avoidance and aggression appears to be consistent across various populations, ages, and types of aggression.

Existing research also highlights the role of moral absolutism, a form of cognitive rigidity, in the perpetration of IPV. Individuals with rigid moral beliefs often justify violent behavior as a response to perceived moral transgression by their partner, engaging in self-deception to maintain a sense of righteousness (Vecina et al., 2016). A study by Romero-Martínez et al. (2016) found that court-mandated intervention programs improved empathy and cognitive flexibility in IPV perpetrators, but individuals with high alcohol consumption showed a smaller gain and a higher risk of repeat violent behavior. Similarly, Vitoria Estruch et al. (2017) found that cognitive rigidity interacts with substance use, as individuals with high mental rigidity and alcohol use were significantly more likely to perpetrate IPV, struggling to regulate emotions or de-escalate conflicts. These findings underscore the need for interventions targeting cognitive flexibility to reduce IPV risk.

### 1.2. Acceptance and Commitment Therapy-Based BIP

A specialized adaptation of acceptance and commitment therapy (ACT) designed to address violent behavior, particularly in individuals who have engaged in IPV, has recently shown promise in reducing criminal behavior and recidivism (e.g., Zarling et al., 2019, 2020). This ACT-based BIP integrates the core principles of ACT, such as promoting psychological flexibility and reducing psychological inflexibility, with specific strategies tailored to reduce aggression and violent behavior. This program has become the standard treatment model in several locations across the United States for BIPs in community corrections. Research has compared the effectiveness of this ACT-based BIP to traditional BIP programming and victims of ACT participants reported experiencing significantly fewer IPV behaviors compared to victims of participants in traditional BIP programming (Zarling & Russell, 2022).

Despite these promising findings, there is a lack of evidence on the changes that occur during ACT-based BIP that may contribute to its success. While some studies have explored changes in emotion regulation and experiential avoidance following ACT-based treatments for violent individuals, there have been mixed results. Some have documented decreases in experiential avoidance and maladaptive emotion regulation (Zarling et al., 2015), whereas others have reported no significant changes (Malouf et al., 2017). These mixed findings underscore the need for further research to clarify the mechanisms driving change in ACT-based interventions.

### 1.3. Current Study

The primary aim of this study is to evaluate changes in psychological flexibility and IPV over time during the intervention period for men participating in a court-mandated domestic violence intervention program (ACT). Specifically, this study seeks to determine whether clients show significant increases in psychological flexibility across three assessment points (time one, time two, and time three) and whether IPV behaviors decrease significantly over the same period. Consistent with the theory underlying ACT, it is hypothesized that psychological flexibility will significantly improve over the assessment period and that IPV behaviors will significantly decrease across the three time points.

Additionally, this study explores the relationship between psychological flexibility and IPV at each time point, hypothesizing that higher levels of psychological flexibility will be associated with lower levels of IPV. A key focus of the analysis is to investigate whether changes in psychological flexibility can predict reductions in IPV. It is hypothesized that psychological flexibility at time three will significantly predict IPV at time three, even after controlling for IPV at time one, age, and race, with psychological flexibility explaining a meaningful proportion of the variance in IPV outcomes.

## 2. Methods

### 2.1. Participants

Participants in the current study included men who were court-mandated to the Iowa Domestic Abuse Program (IDAP) after being convicted of a domestic assault charge against a female partner. The IDAP classes included in the current study were taking place in community-based corrections and utilized the ACT curriculum. The sample (*N* = 56) of clients was all male and ranged in age from 19 to 60 years old with a mean of 34.49 (*SD* = 9.31). Clients were mostly White (56.60%) and African American (33.96%) (see Table 1).

### 2.2. Procedure

ACT facilitators were initially contacted via email to determine their group’s interest and availability to participate. Of the 20 facilitators initially contacted, 8 agreed to have their group be included in the current study. Once a facilitator expressed interest, a research staff member explained the study procedures and then asked to schedule a time to attend one of their ACT group sessions within the following month. This attendance was arranged for the end of a group session to minimize disruptions to the program’s standard format. At the conclusion of the scheduled group session, the facilitator exited the room or Zoom call to ensure confidentiality and reduce any potential influence on client decision-making. The research staff member then introduced the study to the clients, emphasizing its voluntary nature and assuring them that their decision to participate would remain anonymous whether they participated or not. For virtual groups, the survey link was shared via the chat feature, while for in-person groups, a QR code was provided. Participants who chose to participate were directed to an informed consent document where they could decline or continue with the surveys. They were then invited at two subsequent time points over the next two months. The time period between surveys was an average of 4 weeks, with the average time span from time one to time three being approximately 2 months. The length of time between each survey varied across participants. Because of absences or group cancellations, it ranged anywhere from 2 weeks to 5 weeks between survey administrations. Group size varied from 8 to 17 participants with an average of 6 men in each group agreeing to take part in the current study.

#### ACT-Based BIP

The ACT program is a strengths-based, trauma-informed intervention designed for men in Iowa’s community-based domestic abuse programs, rooted in the principles of acceptance and commitment therapy (ACT). Program requirements include attendance at 24 sessions, which are held once a week for 90 min. Facilitators engage participants from an accepting, defused, and value-directed stance, using experiential learning to encourage present-moment awareness, avoid struggle, and view resistance as a natural part of the change process. Sessions center on experiential exercises like the Matrix, a tool based on relational frame theory (RFT; Hayes et al., 2001) and functional contextualism (Gifford & Hayes, 1999). The Matrix helps participants notice and sort aspects of their experiences, differentiating between sensory and mental experiences while categorizing behaviors as “toward moves” (aligned with values) or “away moves” (aimed at avoiding unwanted thoughts or feelings). This interactive tool simplifies the application of ACT concepts, ensuring focus on skill-building rather than distractions like lecturing or excessive problem-solving.

Core components include awareness of internal experiences, learning new ways to respond to emotions, stepping back from problematic thoughts, and distinguishing value-driven behaviors from avoidance behaviors. Relationship skills are cultivated through role plays and practice of respectful, healthy communication behaviors. Specific topics are tailored to the population, such as masculinity (defusing from rigid gender norms), fatherhood (identifying desired parenting approaches), trauma history (awareness and acceptance of adverse experiences), and substance use (its influence on behavior and values). The ACT program ultimately aims to equip participants with skills to live healthier, value-driven lives and foster meaningful, lasting behavioral change.

### 2.3. Measures

Psychological Inflexibility. Client psychological inflexibility was assessed using the 23-item Comprehensive Assessment of Acceptance and Commitment Therapy processes (CompACT; Francis et al., 2016). The scale contains the three subscales of openness to experience, behavioral awareness, and valued action. The scale contains negatively valenced items such as “I tell myself I shouldn’t have certain thoughts” and “I work hard to keep out upsetting feelings” as well as positively valenced items such as “I can take thoughts and feelings as they come without attempting to avoid them” and “My values are reflected in my behavior”. Of note, the scale was developed with social desirability in mind, which can be a concern in forensic populations, and was found to have excellent discriminant validity (correlations ranging from −0.01 to 0.03). Cronbach’s alpha was 0.91 in Francis et al. (2016). In the current study, Cronbach’s alpha was 0.99 at time one, 0.99 at time two, and 0.99 at time three.

Intimate Partner Violence. Thirteen items representing the Composite Abuse Scale Revised–Short Form psychological and physical abuse subscales were administered at all three timepoints (CASR-SF; Ford-Gilboe et al., 2016). The CAS_R_-SF was originally developed as a self-report measure for use with victims, but the current study modified the scale to be used with perpetrators and to measure change from session to session. The instructions were modified to “Since last session, how often have you …”, and response options were truncated to 1 = never to 4 = daily or almost daily. Participants were asked to rate how often they have engaged in a variety of abusive behaviors—for example, “Shook, pushed, grabbed or threw my partner”, “Told my partner they were crazy, stupid or not good enough”, and “Blamed my partner for my behavior”. Cronbach’s alpha was 0.80 at time one, 0.66 at time two, and 0.64 at time three in our study, and 0.94 in Ford-Gilboe et al. (2016).

### 2.4. Analytic Plan

Data were prepared and analyzed in Stata version 17 and SPSS version 29. Composite scores were computed by taking the mean of non-missing scores. Descriptives were examined, and correlations were performed. Time one IPV scores were found to have kurtosis (2.33, *SD* = 0.63), so scores were winzorized at two standard deviations above the mean (1.01) due to right skew, affecting two values and resulting in a kurtosis statistic within the acceptable range (−0.06, *SD* = 0.63). The mean of IPV at time one without winzorizing was 0.44 (*SD* = 0.28), and the winzorized mean, used below, was 0.43 (*SD* = 0.25). Changes in client psychological inflexibility and IPV over time were evaluated with repeated measures ANOVAs and follow-up paired *t*-tests. A regression predicting client IPV at time three was performed with psychological inflexibility as the primary predictor and IPV at time one, age, and race as control variables. The categories of race were collapsed due to the number of participants in some categories. Race was entered into the regression with White as the reference, an indicator for African American or Black, and an indicator for all other races, reflecting White–Hispanic, Black–Hispanic, and Asian clients. Semi-partial *r^2^* was examined as a measure of effect size.

## 3. Results

Psychological inflexibility was highly correlated with itself across time, e.g., *r* = 0.96, *p* < 0.01 between time one and two. IPV was less highly correlated, e.g., *r* = 0.79, *p* < 0.01 between time one and two. Correlations between client psychological inflexibility and IPV were moderate, e.g., *r* = 0.75, *p* < 0.01 at time one for both constructs (see Table 2).

Average client psychological inflexibility improved at each time point. The mean at time one was 3.90 (*SD* = 1.18). The mean at time two was 3.63 (*SD* = 1.13), and the mean at time three was 3.28 (*SD* = 1.12). In a repeated measures ANOVA, Mauchly’s test of sphericity was significant, so Greenhouse–Geisser statistics are reported. The change was significant (*F* (1.14) = 34.56, *p* < 0.01, *partial eta squared* = 0.46). Follow-up paired *t*-tests showed time one was significantly different from time two (*t* (44) = −4.92, *p* < 0.01, Cohen’s *d* = −0.73). Time two was significantly different from time three (*t* (41) = −6.15, *p* < 0.01, Cohen’s *d* = −0.95), and time one was significantly different from time three (*t* (41) = −6.06, *p* < 0.01, Cohen’s *d* = −0.94). These differences had medium to large effect sizes.

The average number of IPV behaviors decreased at each time point. The mean at time one was 0.43 (*SD* = 0.25); time two was 0.31 (*SD* = 0.19); and time three was 0.19 (*SD* = 0.15). The repeated measures ANOVA violated the assumption of sphericity, and Greenhouse–Geisser statistics are reported. This change was significant (*F* (1.52) = 39.42, *p* < 0.01, *partial eta squared* = 0.49). Follow-up paired *t*-tests showed significant differences between time one and time two (*t* (44) = −5.38, *p* < 0.01, Cohen’s *d* = −0.80), time two and three (*t* (41) = −5.05, *p* < 0.01, Cohen’s *d* = −0.78), and time one and time three (*t* (41) = −7.10, *p* < 0.01, Cohen’s *d* = −1.10). Effect sizes were medium to large.

### Regression Analysis

Regression analysis was performed with psychological inflexibility predicting IPV at time three with IPV at time one, age and race as controls. CompACT was a significant predictor of time three IPV (*B* = 0.06, *SE B* = 0.02, *p* < 0.01, *β* = 0.47, *semi-partial r*^2^ = 0.15), explaining 15% of the variance in the outcome. Time one client IPV did not significantly predict time three IPV. Client age and race also were not significant predictors. The model was significant overall (*F* (5, 36) = 5.81, *p* < 0.01, *R*^2^ = 0.45) with 45% of the variance in time three IPV explained by the model (Table 3).

## 4. Discussion

The present study evaluated changes in psychological inflexibility and intimate partner violence (IPV) behaviors over time among men participating in a court-mandated domestic violence intervention program using the ACT curriculum. Findings demonstrated significant decreases in psychological inflexibility and corresponding decreases in IPV behaviors across the three assessment points. Effect sizes for these changes were medium to large at each interval, indicating substantial improvements. Psychological inflexibility and IPV behaviors were positively correlated at all time points, aligning with the hypothesized relationship. Furthermore, regression analysis revealed that psychological inflexibility at time three significantly predicted IPV at time three, even after controlling for baseline IPV, age, and race. Psychological inflexibility explained 15% of the variance in IPV outcomes, underscoring its critical role as a mechanism of change. To our knowledge, it has not previously been tested whether decreases in psychological inflexibility during the intervention are linked to decreases in IPV. Hence, our findings extend earlier research.

The observed decreases in psychological inflexibility over time suggest that the ACT program effectively targets processes such as openness to experience, behavioral awareness, and valued action. These processes are central to the ACT framework, which emphasizes fostering acceptance and mindfulness while encouraging committed actions aligned with personal values. By cultivating these abilities, participants may develop healthier coping mechanisms and greater emotional resilience, reducing the likelihood of engaging in abusive behaviors. The size of changes in psychological inflexibility appears commensurate with other studies examining changes in psychological flexibility over the course of treatment (e.g., Benoy et al., 2019; Kohtala et al., 2018).

The corresponding decreases in IPV behaviors further support the theory that improvements in psychological inflexibility facilitate behavior change. Participants who became more psychologically flexible may have been better equipped to manage emotional triggers, reduce experiential avoidance, and engage in non-violent, value-driven behaviors. This finding highlights the potential of ACT-based interventions to address the underlying mechanisms driving IPV, adding to the evidence that experiential avoidance of negative emotional reactions helps to motivate abusive and aggressive responses within difficult relationship situations (e.g., LaMotte et al., 2025). Notably, psychological inflexibility emerged as a significant predictor of IPV outcomes at time three, even after accounting for baseline IPV and demographic factors. This result underscores its role as a potential mechanism of change in reducing abusive behaviors. The lack of significant predictive effects for age and race suggests that the intervention’s effectiveness may be consistent across demographic subgroups, highlighting the potential for broad applicability.

The findings of the current study align with prior studies demonstrating the efficacy of ACT-based interventions in promoting behavioral and emotional regulation. Previous research has shown that reductions in experiential avoidance and improvements in mindfulness are associated with decreased aggression (Garofalo et al., 2020; Navas-Casado et al., 2023). This study builds on this body of work in the literature by applying ACT principles to a forensic population within a court-mandated program, providing evidence for its utility in this unique context.

This study also addresses a gap in the literature regarding the mechanisms of change in IPV interventions, reflecting the growing focus within the research community on uncovering the specific mechanisms—such as program components, individual traits, and program outcomes—that effectively reduce the likelihood of repeat criminal behavior or violence (e.g., Serin et al., 2013). Little is known about what factors are the most productive targets of change in BIPs. While many evaluations of BIPs focus solely on outcomes, this study highlights the importance of targeting and measuring psychological inflexibility as a mechanism of change. These results contribute to the growing evidence base supporting the integration of ACT principles into interventions for IPV (e.g., Zarling et al., 2019, 2020; Zarling & Russell, 2022).

### 4.1. Strengths and Limitations

This study’s strengths include its longitudinal design, which allowed for the assessment of changes over time, and the rigorous statistical analyses used to evaluate key relationships. Despite its strengths, this study has limitations that warrant consideration. The small sample size may limit statistical power and the generalizability of findings. Self-report measures, particularly in a forensic population, are susceptible to response biases. Social desirability or lack of attentiveness may have influenced the observed correlations. Given that the CompACT includes items that are positively worded and capturing traits such as emotional resilience and openness, participants may have been inclined to endorse higher scores on this measure while underreporting violence behaviors. We also did not assess the number of participants with active no contact orders at the time of the intervention, which could have influenced opportunities to engage in IPV.

Identifying the best timing and frequency of observations to capture critical points of change is especially complex without prior knowledge of the pace or nature of change. In the current study, the length of time between each time point varied and was not consistent across participants. Because of absences or group cancellations, it ranged anywhere from 2 weeks to 5 weeks between survey responses. Researchers must balance effective study design with minimizing participant burden and avoiding artifacts from excessive data collection. Evidence suggests change often occurs in sudden moments rather than gradual, linear change. Assessing these shifts and their temporal relationship to outcomes has been particularly difficult to capture in previous studies, and this study is no different.

Furthermore, demonstrating causality in mechanisms of change within BIPs is challenging, even in studies designed for this purpose. Advancing research on BIPs requires refining methods to pinpoint the active ingredients driving change. Without a control group, we are not able to make definitive conclusions regarding changes in psychological flexibility and IPV that may occur due to the passage of time versus a result of the ACT intervention. In an era of constrained resources for corrections, the ability of treatment providers to sustain support for evidence-based interventions depends on clearly demonstrating their effectiveness in reducing risk. The current study’s results, while promising, highlight the need for continued investigation into how psychological flexibility and other dynamic risk factors interact with program elements to produce lasting behavioral change. By advancing our understanding of these mechanisms, researchers and practitioners can better align interventions with theoretical models, ensuring that BIPs achieve their intended outcomes while maximizing resource efficiency.

### 4.2. Implications and Future Directions

The findings have important implications for the refinement and implementation of ACT programs for court-mandated populations. Targeting psychological inflexibility appears to be a promising approach for reducing IPV behaviors, highlighting the potential for ACT-based interventions to address the underlying mechanisms of abusive behavior. These interventions could also be adapted for broader populations or prevention-focused settings, expanding their reach and impact.

Future research should replicate this study with larger, more diverse samples to enhance the generalizability of findings. Additional moderating or mediating variables, such as trauma history or substance use, should be explored to better understand the relationship between psychological flexibility and IPV outcomes. Longitudinal studies with extended follow-up periods are needed to examine the durability of changes in psychological flexibility and IPV behaviors. Finally, incorporating mixed-method approaches could provide valuable qualitative insights into client experiences and program engagement, complementing quantitative findings.

## Figures and Tables

**Table 1:** Demographic characteristics.

Variable	*n* or *m*	*%* or *SD*
Age (range = 19 to 60)	34.49	9.31
Race/Ethnicity		
White	30	56.60%
Black or African American	18	33.96%
Other	5	9.43%

Note. *N* = 53. The race category “other” includes White–Hispanic, Black–Hispanic, and Asian due to small n.

**Table 2:** Psychological flexibility and IPV over time: means and correlations.

Variable	*m*	*SD*	*n*	1	2	3	4	5
1. CompACT Time 1	3.90	1.18	56					
2. CompACT Time 2	3.63	1.13	45	0.96 **				
3. CompACT Time 3	3.28	1.12	42	0.84 **	0.94 **			
4. IPV Time 1	0.43	0.25	56	0.75 **	0.64 **	0.49 **		
5. IPV Time 2	0.31	0.19	45	0.69 **	0.58 **	0.50 **	0.79 **	
6. IPV Time 3	0.19	0.15	42	0.41 *	0.48 **	0.59 **	0.49 *	0.61 **

* *p* < 0.05, ** *p* < 0.01.

**Table 3:** Regression analysis predicting time three client IPV.

Variables	*B*	*SE B*	*β*	*Semi-Partial r* ^2^
CompACT	0.06 **	0.02	0.47	0.15 **
Time 1 IPV	0.12	0.09	0.23	0.03
Age	<−0.01	<0.01	−0.03	<0.01
Race (White as the reference)				
Black or African American	−0.03	0.04	−0.10	0.01
Other	0.09	0.08	0.16	0.02

Note. *N* = 42. The race category “other” includes White–Hispanic, Black–Hispanic, and Asian due to small n. ** *p* < 0.01.

## Data Availability

Data is contained within the article.
